# Catastrophic Impact Loading Resilience of Welded Joints of High Strength Steel of Refineries’ Piping Systems

**DOI:** 10.3390/ma15041323

**Published:** 2022-02-11

**Authors:** Andrzej Klimpel, Anna Timofiejczuk, Jarosław Kaczmarczyk, Krzysztof Herbuś, Massimiliano Pedot

**Affiliations:** 1 The Department of Welding, The Faculty of Mechanical Engineering, The Silesian University of Technology, Konarskiego 18A, 44-100 Gliwice, Poland; andrzej.klimpel@polsl.pl; 2 The Department of Fundamentals of Machinery Design, The Faculty of Mechanical Engineering, The Silesian University of Technology, Konarskiego 18A, 44-100 Gliwice, Poland; anna.timofiejczuk@polsl.pl (A.T.); massimiliano.pedot@polsl.pl (M.P.); 3 The Department of Theoretical and Applied Mechanics, The Faculty of Mechanical Engineering, The Silesian University of Technology, Konarskiego 18A, 44-100 Gliwice, Poland; jaroslaw.kaczmarczyk@polsl.pl; 4 The Department of Engineering Processes Automation and Integrated Manufacturing Systems, The Faculty of Mechanical Engineering, The Silesian University of Technology, Konarskiego 18A, 44-100 Gliwice, Poland

**Keywords:** refinery, piping, welded joint, API 5L X80 steel, nonlinear strength analyses, FEM

## Abstract

Refineries piping installation systems are designed, fabricated, and operated to assure very high levels of quality and structural integrity, to provide very high resilience to catastrophic events like earthquakes, explosions, or fires, which could induce catastrophic damage of piping systems due to collapse of nearby structures as towers, bridges, poles, walkways, vessels, etc. To evaluate the catastrophic impact loading resilience to failure of MMA (Manual Metal Arc Welding), GMA (Gas Metal Arc Welding), SSA (Self-shielded Arc Welding), and LASER+GMA of modern API 5L X80 pipes butt welded joints used for piping installation systems of refineries, the new, original technique of the quantitative and qualitative evaluation of impact loading resilience of butt welded joints of pipes was developed. The high-quality butt welded joints were impact loaded by the freely dropping 3000 kg mass hammer of the die forging hammer apparatus. The impact loading energy needed to exceed the yield strength of the extreme zone of welded joints and to induce catastrophic fracture of butt welded joints of API 5L X80 pipes was calculated using FEM (Finite Element Method) modeling of the impact loading process of tested butt welded joints of pipes. Results of the FEM modeling of impact loading technique of butt welded joints of piping systems indicate that it is a useful tool to provide valuable data for experimental impact loading tests of welded joints of pipes, decreasing the time and cost of the experiments. The developed impact loading technique of butt welded joints of pipes to simulate the catastrophic events in refinery piping systems and evaluate the resilience of the butt welded joints of pipes to catastrophic failure proved to be very efficient and accurate. Experiments of impact loading indicated that all specimens of butt welded joints API 5L X80 steel pipes are resilient to failure (cracks) in the extreme stressed/strained areas, above yield and tensile strength of the weld metals, no cracks or tears appeared in the extreme stressed/strained areas of the edges of the pipes, proving the very high quality of API 5L X80 steel pipes.

## 1. Introduction

**Figure 1:**
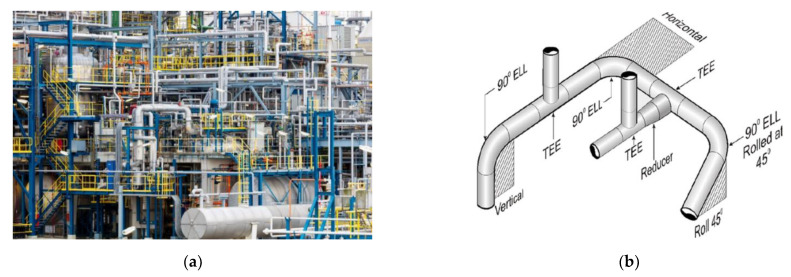
Example of the piping systems of refinery plants (**a**) A view of the magnitude of piping installations required in Jamnagar India refinery, the world’s largest oil refinery with an aggregate capacity of 1.24 million barrels [7,8,9]; (**b**) A view of typical elements of the refinery piping installation systems where pipes butt joints are welded in horizontal–PC (2G) and vertical position–PH (5G) [7,8,9].

Piping systems within refinery companies enable the continuous transfer of raw materials for the purposes of the assumed technological process of crude oil processing. Therefore, the condition of these systems directly impacts the safe operation of the company and ensures the required efficiency of the technological process. The piping systems of refineries include, among others, linear pipe sections, various types of pipe fittings, devices for forcing the circulation of the raw material (pumps), elements controlling its flow direction (valves), heat exchange systems, and systems for collecting the product. Welded joints are also considered the main parts of piping systems and as elements that are critical to the safe operation of refineries (Figure 1) [1,2,3,4,5,6,7,8,9,10]. The initial stage of the piping system design process is to define the functional requirements for the geometric form of its route. Meeting these assumptions will enable obtaining a safe transport route of the raw material from the starting point to the endpoint. On the other hand, its final form is influenced by such factors as the type of the transported raw material, the speed and the flow rate, the working pressure, the ambient temperature and the temperature of the transported fluid, results of the selection of design features based on strength analyses [4,5,6].

In order to determine safe values of the design features of the designed and manufactured pipe systems, with particular emphasis on welded joints, in addition to the previously mentioned values, the possibility of catastrophic events should be considered, such as the destruction of coexisting elements of the company’s infrastructure. Items such as tanks, poles, or parts of a sidewalk falling onto the piping can be responsible for causing catastrophic impact loads. In addition, to ensure a very high level of resistance to catastrophic events, such as earthquakes, explosions, or fires, refinery piping systems are designed to provide a high level of integrity of the geometric form of their structure, with particular emphasis on preventing their unsealing [7,10]. The occurrence of a catastrophic situation causes an increase in the occurrence of dangerous situations for the company’s staff, a break in the operation of the refinery infrastructure, and the necessity to carry out the process of diagnostics for damages and their removal. Critical areas of the piping system design process are related to the design of various types of connections, such as: flanged or welded. In relation to welded joints, it is very important to search for knowledge on the influence of possible impact loads on their properties. The data obtained in this way can be used to design specific structural nodes of future refinery piping systems.

One of the basic factors describing the failure of piping systems is the Pipe Diameter Factor (PDF). The PDF describes the relations between the pipe diameter and the possible severity of the failure. As the diameter of the pipe increases, the possible severity of failure increases. The PDF factor for a pipe of 12.0 inches (304 mm) in diameter is higher than for a pipe of 8.0 inches (203.2 mm). As the most modern and typical solution of piping systems, API 5L X80 high strength steel pipes 323.9 mm (12.75 inches) dia. and wall thickness 10.0 mm, HF longitudinally welded, produced by HUTA ŁABĘDY S.A. Poland were selected, and four welding processes were used to create specimens for catastrophic impact loading tests: MMA, GMA, SSA, and LASER + GMA. The research results presented in the paper are a continuation of research related to the quasi-static loading of the same welded joints of API 5L X80 steel pipes [11].

The following study was done to simulate and experimentally test the catastrophic impact loading resilience of the high-quality MMA, GMA, SSA, and LASER+GMA butt welded joints of API 5LX80 pipes of refineries piping systems:

- FEM modeling of the impact loading process of welded joints to establish the value of kinetic energy (the hammer mass and the hammer height of the forging hammer apparatus) of catastrophic load to provide the level of stresses and strains of the welded joint extreme stressed/strained areas, above the yield stress level of the parent material–API 5L X80 pipes and the weld metals to initiate cracks of the butt welded joints and parent material as well.
- Newly developed catastrophic impact loading tests of the butt welded joints specimens under impact energy calculated by FEM modeling to evaluate the resilience to impact loading and quality of welded joints of API 5L X80 pipes.

## 2. Preparation of Specimens of Butt Welded Joints of Pipes

The Welding Procedure Specifications (WPS) were worked out in Mostostal S.A. Zabrze, Poland, to prepare the butt welded joints specimens of sections of 120 mm width of API 5LX80 steel pipes 323.9 mm dia. and wall thickness 10.0 mm for catastrophic impact loading experiments (Table 1). 

The welding conditions of three basic welding processes commonly used in the production of piping systems of refinery plants: MMA, GMA, and SSAW, and one, the most modern solution pipes’ butt joints welding techniques–root pass laser welded and filling and cap passes—GMA welded, are presented in Table 2. The welding conditions of three basic welding processes commonly used in the production of piping systems of refinery plants: MMA, GMA, and SSAW, and one, the most modern solution pipes butt joints welding techniques–root pass laser welded and filling and cap passes—GMA welded, are shown in Table 2. The welding consumables (filer metals) were used for welded joints’ specimens, assuring similar mechanical properties to the API 5L X80 high strength steel pipes, as shown in Table 1 and Table 2.

## 3. Results of FEM Nonlinear Analysis of the Impact Loading Process of MMA, GMA, SSA, and LASER+GMA Butt Welded Joints of API 5LX80 Pipes 

### 3.1. Material to Be Studied

**Figure 2:**
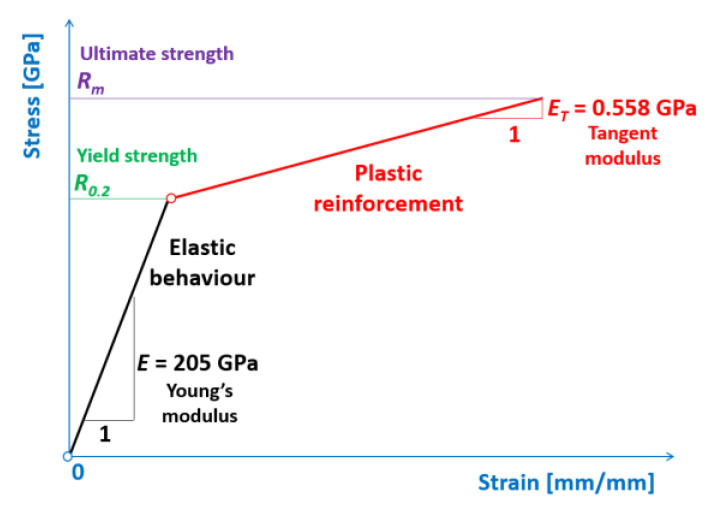
Bilinear elastic-plastic API 5L X80 steel pipes material model.

The bilinear elastic-plastic material model of a welded joint of API 5L X80 steel pipes was adopted for performing the numerical simulations, as presented in Figure 2. 

In Table 1 and Table 3, the details of the weld metals and API 5L X80 steel pipes material properties for which the numerical calculations were performed are juxtaposed. The assumed bilinear material model belongs to the simplest but shows a nonlinear stress-strain behavior.

### 3.2. Physical Model of the Butt Welded Joint of Pipes Specimens

**Figure 3:**
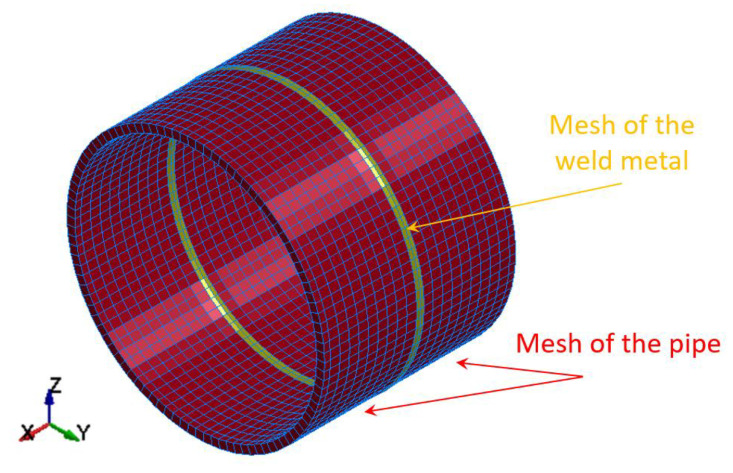
Discretization of the arc butt welded pipes into finite elements.

The physical model of the MMA, GMA, SSA, LASER+GMA butt welded joints of sections of 120 mm width of API 5L X80 steel pipes, dia. 323.9 mm and wall thickness 10.0 mm, impact loaded in the die forging hammer apparatus is shown in Figure 3. The mean thickness of the welded joints reinforcement is 12.0 mm, and the mean width of the weld metal is 9.0 mm (the width of the weld face is approximately 16.0 mm, and the width of the weld root face is approximately 2.0 mm). Because the welded joints’ HAZ (Heat Affected Zone) is very narrow, it was assumed to treat the HAZ as part of the weld metal. The butt welded joints of the pipes specimen have been divided into shell finite elements with five degrees of freedom in a node. Five Gauss integration points on the thickness of the shell of the butt welded joints of pipes were assumed. The weld metals and the pipes were mutually connected using the same nodes (without introduced contact) because they formed one inseparable entity.

**Figure 4:**
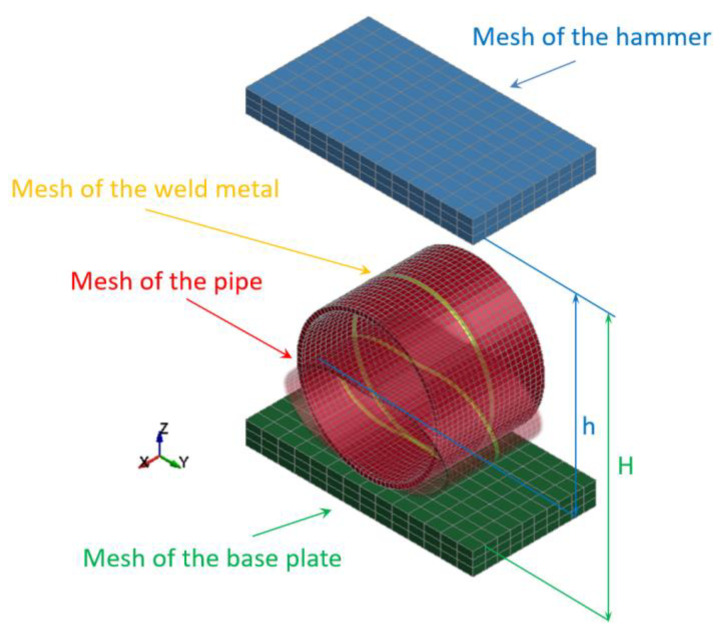
Discretization of the butt welded joint of pipes (a specimen), the hammer, and the base plate of the die forging hammer into finite elements.

The specimens of butt welded joints of pipes supported by the base plate of the die forging hammer apparatus were impact-loaded by the hammer of the mass 3000 kg. Both hammer and base plate were modeled using solid elements with three degrees of freedom (Figure 4). 

**Figure 5:**
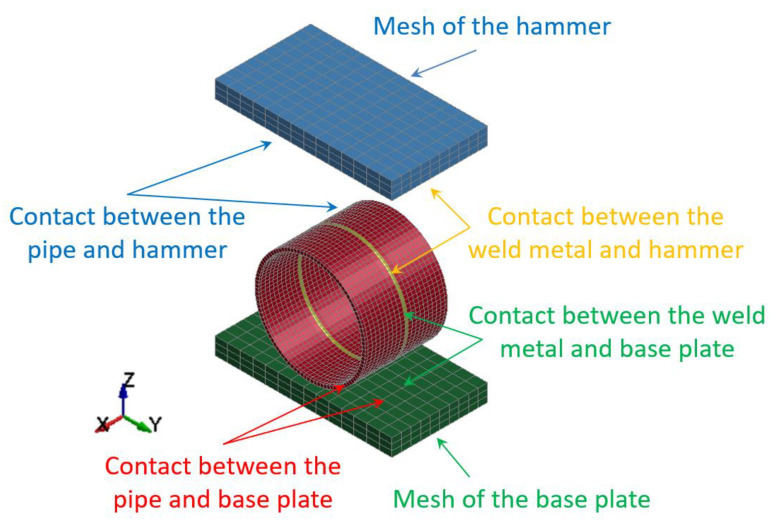
Contact details between modeled welded joint of pipes, the hammer, and the base plate.

When the specimen is placed on the base plate, the hammer is released from a certain height and free falls. Some preliminary cases were analyzed in the hammer height range 0.5 to 1.5 m, but finally, just two optimal cases were analyzed for the height: H = 1.0 m and H = 1.5 m. The distance between the initial hammer position and the top surface of the specimen to be impact loaded (marked as h) was introduced to estimate the potential energy which can be transformed into the plastic deformation of the specimen. The contact between the modeled welded joint of pipes (specimen) and the base plate as well as between the hammer and the specimen was introduced to avoid interpenetrating between the modeled parts (Figure 5).

Coulomb and Moren’s model of friction was considered between all earlier mentioned contact surfaces. The static and kinetic coefficient of friction was assumed as for the steel. The static coefficient of friction equals (µs = 0.15), and the kinetic coefficient of friction was established (µd = 0.1), respectively, for all surfaces being in contact.

The detailed information concerning modeled impact-loaded parts created on the basis of elements and nodes are juxtaposed in Table 4. The most important parts are modeled as deformable (the weld metal and the pipe) because they are the subject of investigation. However, the hammer and base plate are modeled as rigid; therefore, their stiffness is much higher than the stiffness of the specimens of welded joints of pipes.

### 3.3. Numerical Results

**Figure 6:**
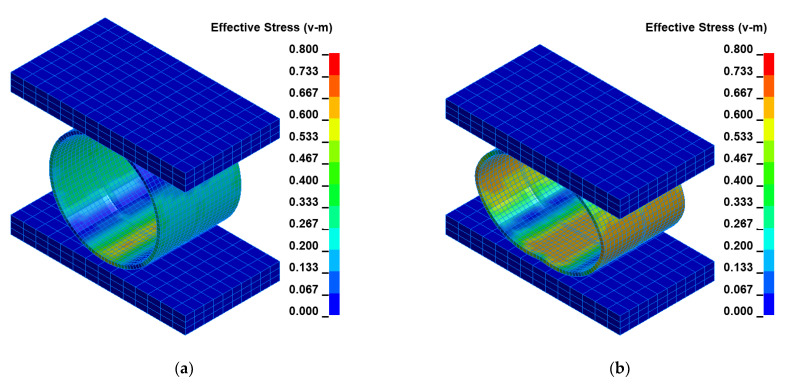

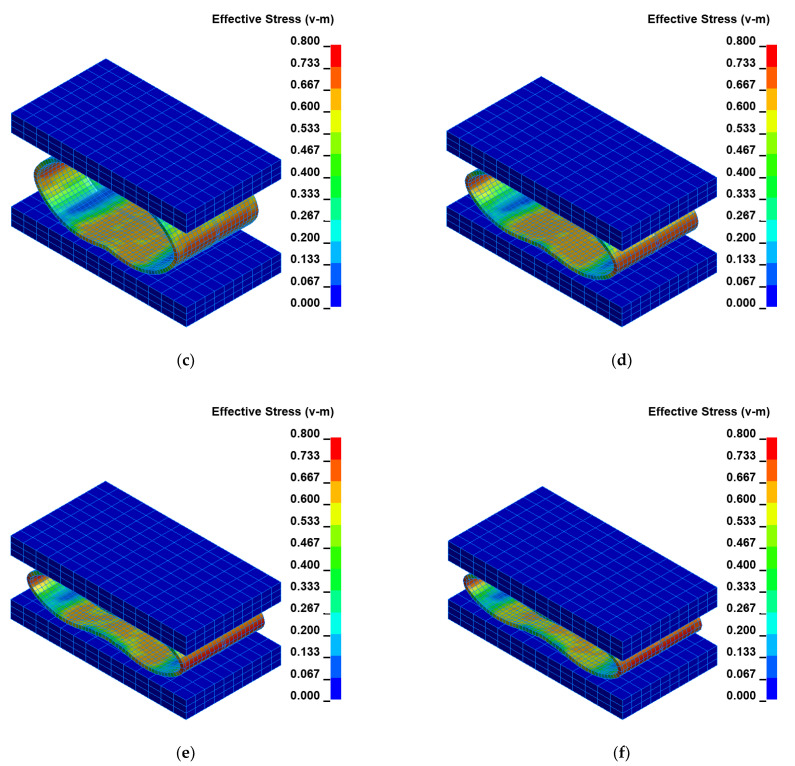
Distribution of the equivalent Huber–Mises stresses [GPa] for arbitrary selected time instants [ms]: (**a**) t = 374, (**b**) t = 394, (**c**) t = 414, (**d**) t = 434, (**e**) t = 454, (**f**) t = 481 for height H = 1.0 m, Table 5.

**Figure 7:**
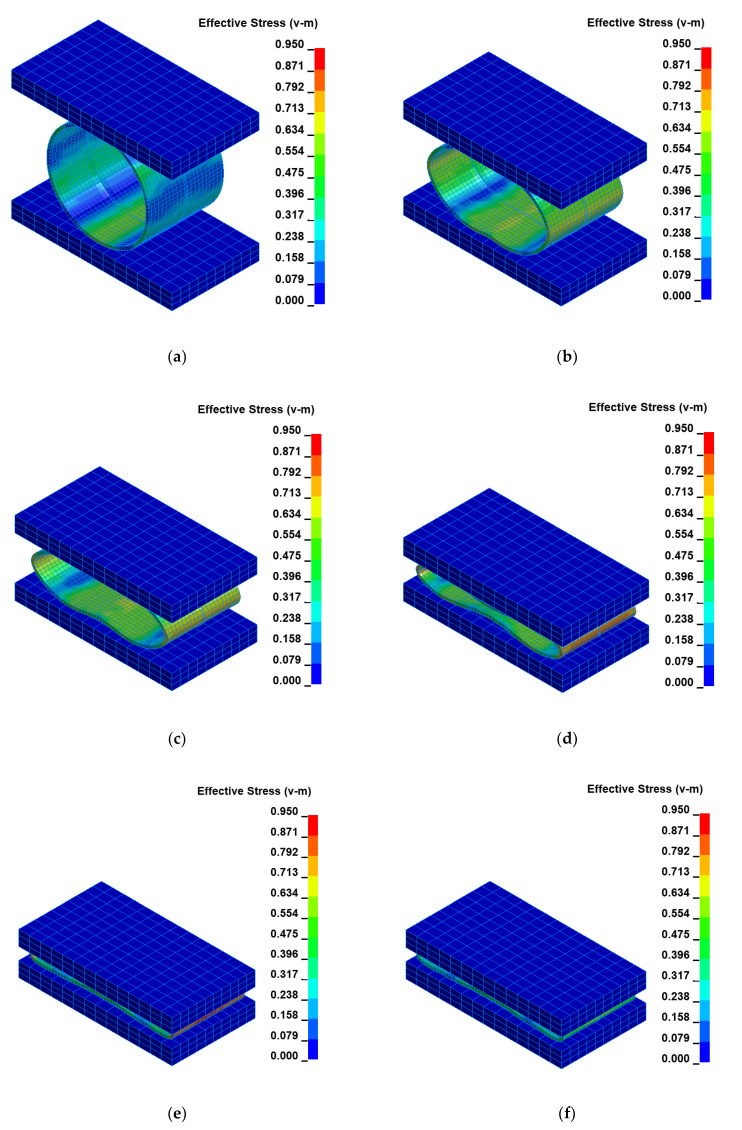
Distribution of the equivalent Huber–Mises stresses [GPa] for arbitrary selected time instants [ms]: (**a**) t = 492, (**b**) t = 512, (**c**) t = 532, (**d**) t = 552, (**e**) t = 572, (**f**) t = 579 for height H = 1.5 m, Table 6.

The impact loading process of the welded joint of API 5L X80 steel pipes was modeled using the finite element method–FEM and computer system LS-DYNA. The numerical calculations results were juxtaposed for several successive time intervals to facilitate the investigation of the mechanism of the process using the impact loading hammer and the base plate of the die forging hammer apparatus. Two optimal variants were researched for two different heights: H = 1.0 m and H = 1.5 m, respectively, as shown in Figure 6 and Figure 7.

To facilitate the analysis of the data shown in Figure 6 and Figure 7, the obtained results were juxtaposed in two tables for two cases for H = 1.0 m and H = 1.5 m, respectively (Table 5 and Table 6). The time and corresponding Huber–Mises stress and effective plastic strain are presented in these tables.

**Figure 8:**
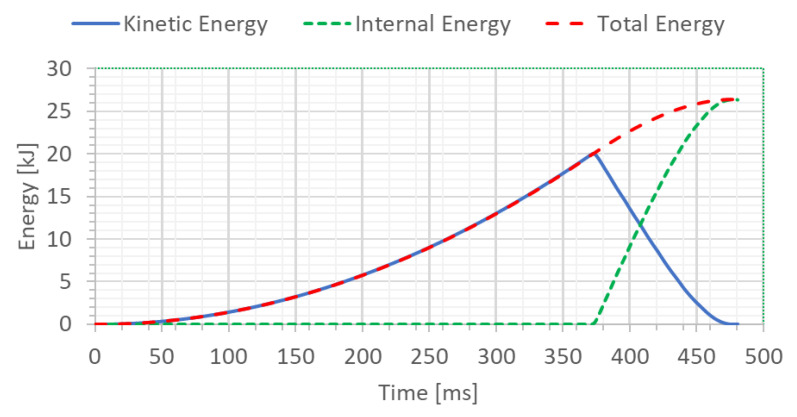
The energy balance [kJ] of the welded joint of pipes for height H = 1.0 m.

**Figure 9:**
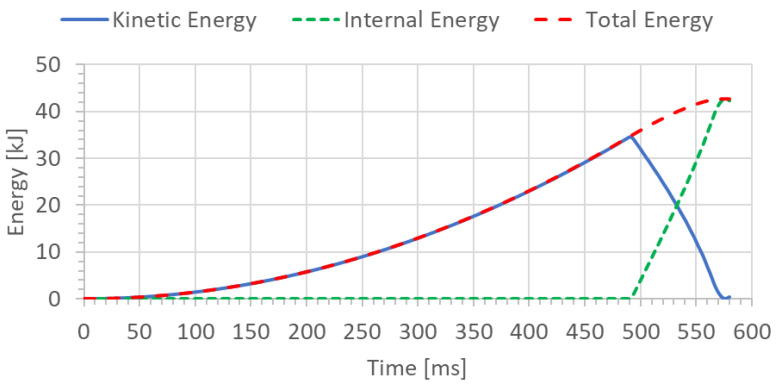
The energy balance [kJ] of the welded pipe for height H = 1.5 m.

To check the correctness of numerical calculations, the energy balance of the welded joint of pipes (specimen) was determined for two analyzed cases, H = 1.0 m and H = 1.5 m, respectively (Figure 8 and Figure 9). The initial potential energy could be compared with the total energy for the final time instant.

The potential energy can be calculated according to the following formula:

(1) $$E_{p}=m\cdot g\cdot h,$$

where:

*m*—mass of the hammer [kg],
*g*—gravitational acceleration [m/s^2^],
*h*—height measured from the initial position of the hammer to the final position of the top surface of the deformed welded joint of pipes [m].

**Figure 10:**
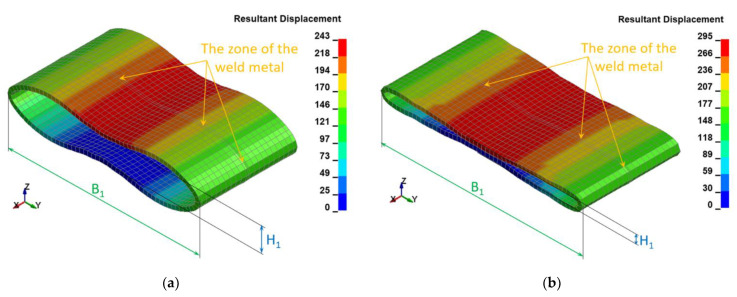
The resultant displacements [mm] in the welded joint of pipes for two variants: (**a**) height H = 1.0 m (H1 = 64 mm; B1 = 461 mm), (**b**) height H = 1.5 m (H1 = 20 mm; B1 = 481 mm).

The mass of the hammer is 3000 kg. The distance between the initial hammer position and the top surface of the deformed welded joint of pipes (marked as *h*) can be found based on the graph (Figure 10) and estimated using, for example, the following formula:

(2) $$h=H+h_{1}-D,$$

where:

*H*—height measured from the initial position of the hammer and the top surface of the base plate [m],
*D*—external diameter of the welded joint of pipes [m],
*h*_1_—displacement of the welded joint of pipes [m] obtained from the graph for the green color (Figure 10); it is given in millimeters, and it should be recalculated into meters.

The potential energy calculated in this manner from Equation (1) can serve to compare it with the total energy or internal energy for the final time instant shown on the graph (Figure 8 and Figure 9) depending on the variant of height (H = 1.0 and H = 1.5 m, respectively).

**Figure 11:**
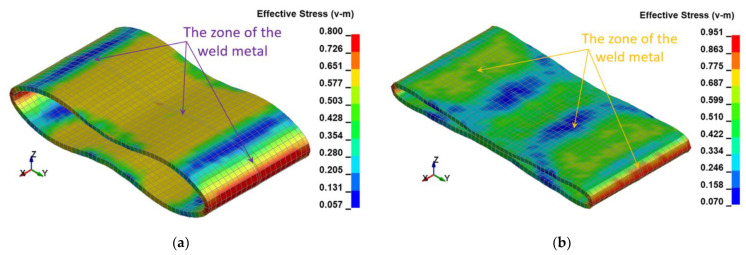
The Huber–Mises stresses [GPa] in the welded joint of pipes for two variants: (**a**) height H = 1.0 m, (**b**) height H = 1.5 m.

**Figure 12:**
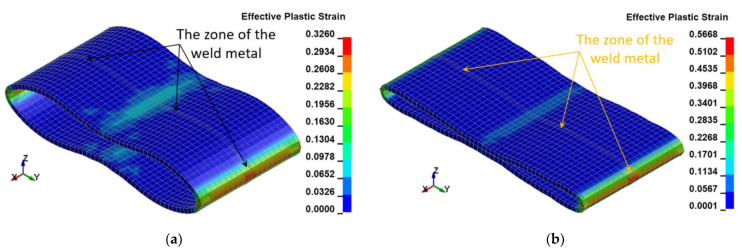
The Huber–Mises effective plastic strains [mm/mm] in the welded joint of pipes for two variants: (**a**) height H = 1.0 m, (**b**) height H = 1.5 m.

**Figure 13:**
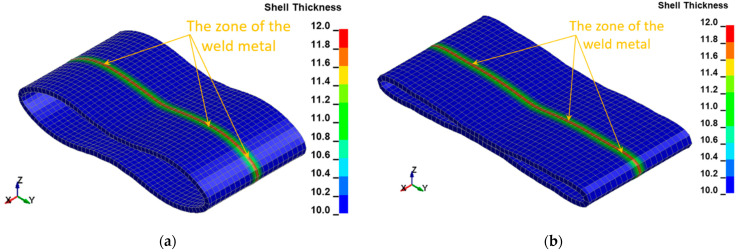
The thickness [mm] of the welded joint of pipes for two variants: (**a**) height H = 1.0 m, (**b**) height H = 1.5 m.

The following resultant values of several selected physical quantities such as displacements (Figure 10), Huber–Mises stresses (Figure 11), effective plastic strains (Figure 12), as well as thickness of the welded pipe (Figure 13) for the final position of the deformed welded joint of pipes are juxtaposed consecutively for two considered variants of height H = 1.0 m and H = 1.5 m, respectively. These data are intended to initially estimate the parameters of the actual experiment.

The FEM modeling analysis indicated that the resultant values for the Huber–Mises Stresses, effective plastic strains, and displacements are much higher in the case of larger height H = 1.5 m, Figure 10, Figure 11 and Figure 12. However, the thickness of the wall of the welded joint of pipes changes insignificantly, and in consequence, this variation can be neglected, as shown in Figure 13. It was estimated that the impact load of the specimen of welded joint of pipes at the hammer height H = 1.0 m induced Huber–Mises stress equal to 800 MPa and effective plastic strain of 0.3256 mm/mm. At the hammer height H = 1.5 m, the impact load-induced Huber–Mises stress equal to 940 MPa and effective plastic strain of 0. 5754 mm/mm. In both cases, the Huber–Mises stress was much higher than the yield strength and tensile strength of MMA, GMA, SSA, and LASER+GMA welded joints of API 5L X800 steel pipes weld metals and parent material, Table 3, Table 5 and Table 6, Figure 6, Figure 7, Figure 8, Figure 9, Figure 10, Figure 11 and Figure 12. 

## 4. Impact Loading Experiments of MMA, GMA, SSA, and LASER+GMA Butt Welded Joints of API 5L X80 Pipes Specimens

**Figure 14:**
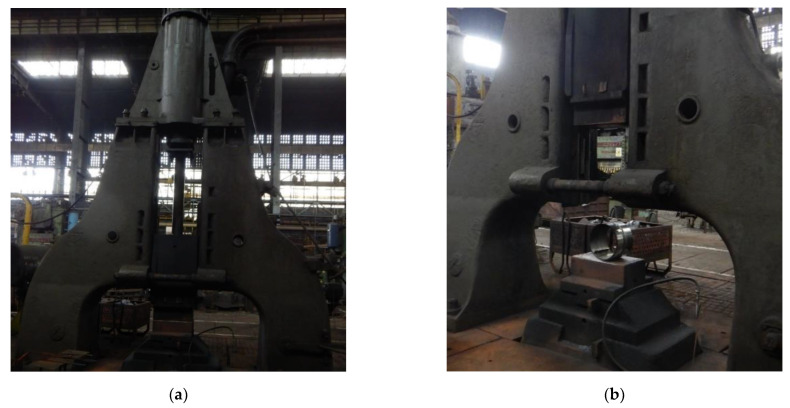
A view of (**a**) the die forging hammer 3000 kg mass, Russian production SKM-3 T apparatus, (**b**) the specimen of MMA butt welded joint of API 5L X80 steel pipes before impact loading test.

**Figure 15:**
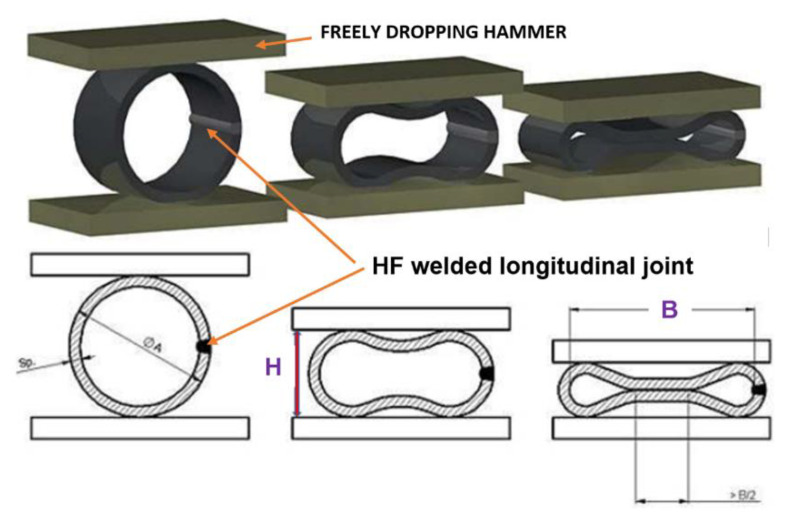
The scheme of impact loading test of butt welded joints of pipes: H and B–geometrical parameters of the deformation [13,14].

**Figure 16:**
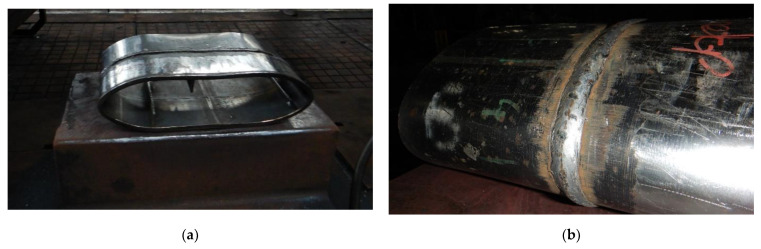
A view of MMA butt welded joint of API 5L X80 steel pipes after the 3000 kg impact loading test–the hammer height 1.0 m. No cracks of both extreme stressed/strained areas of the MMA butt welded joint (**a**,**b**).

The developed impact loading technique to test the resilience to catastrophic impact loading of the MMA, GMA, SSA, and LASER+GMA butt welded joints of API 5L X80 steel pipes was executed at KUZNIA ŁABĘDY S.A. plant (www.kuznia-labendy.pl, accessed on 25 November 2021) on the die forging hammer SKM-3T apparatus (Figure 14), at the load of the 3000 kg weight (mass) of the freely dropping hammer and two hammer heights 1.0 and 1.5 m, selected on the bases of the results of analysis of FEM modeling of impact loading of welded joint of pipes, Table 5 and Table 6, Figure 6, Figure 7, Figure 8, Figure 9, Figure 10, Figure 11 and Figure 12. The scheme of impact loading tests of butt welded joints of API 5L X80 steel pipes is shown in Figure 15. The first impact loading test was done for the MMA butt welded joint specimen at the hammer load of 3000 kg from the height of 1.0 m. The impact loaded specimens were flattened to the geometrical dimensions H and B [mm], and no cracks in extreme stressed/strained areas of the butt welded joint or pipes edges were detected, despite the level of FEM calculated Huber–Mises stresses were over tensile strength of the weld metal and the API 5L X80 steel (Figure 16).

**Figure 17:**
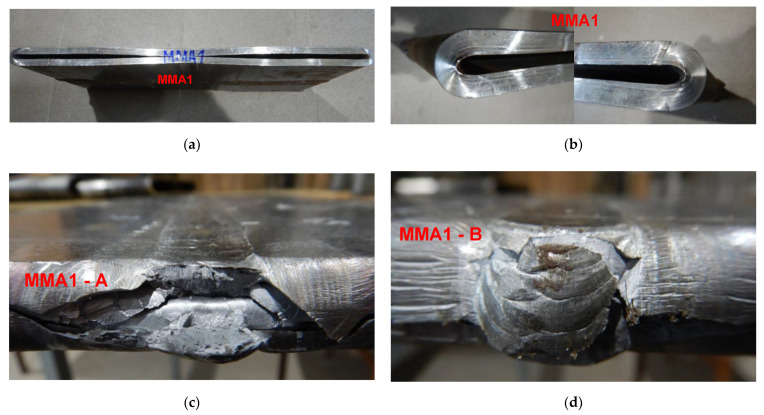
A view of cracked both extreme areas of stressed/strained A and B of MM1 butt welded joint of API 5L X80 steel pipes specimen and pipes edges after second the 3000 kg impact loading test of the specimen of MMA welded joint (the hammer height 1.5 m)–(**c**,**d**). No cracks or tears of the extreme stressed/strained areas of the edges of the pipes (**a**,**b**).

**Figure 18:**
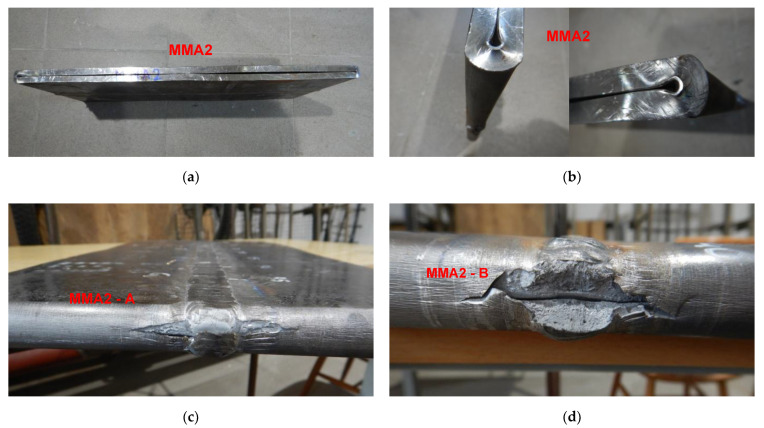
A view of cracked both extreme stressed/strained areas of MM2-A–(**c**) and MM2-B (**d**), of welded joint of API 5L X80 steel pipes specimen and pipes edges after the 3000 kg impact loading test (the hammer height 1.5 m). No cracks or tears of the extreme stressed/strained areas of the edges of the pipes–(**a**,**b**).

**Figure 19:**
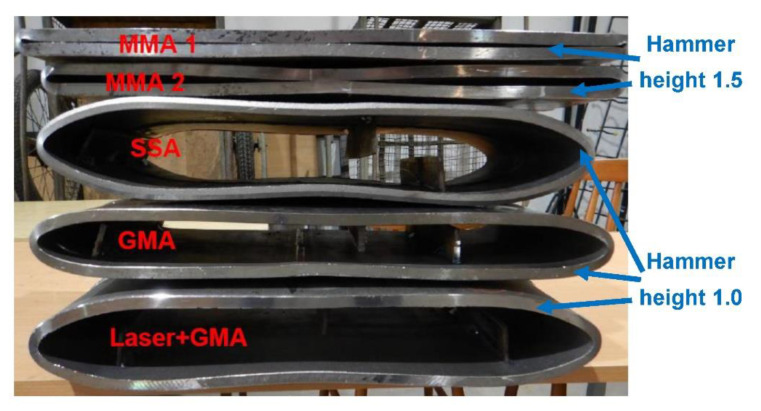
A view of MMA1, MMA2, GMA, SSA, and LASER+GMA butt welded joints of API 5L X80 steel pipes specimens after the 3000 kg hammer impact loading tests (the hammer height 1.0 m and 1.5 m).

**Figure 20:**
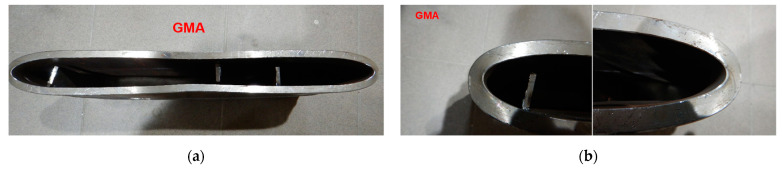

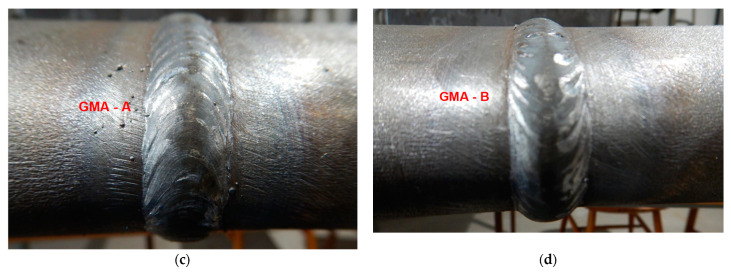
A view of GMA butt welded joint of API 5L X80 steel pipes and pipes edges after the 3000 kg impact loading test (the hammer height 1.0 m). No cracks of both extreme stressed/strained areas of the butt welded joint (**c**,**d**), and no cracks and tears of the extreme stressed/strained areas of the edges of the pipes (**a**,**b**).

**Figure 21:**
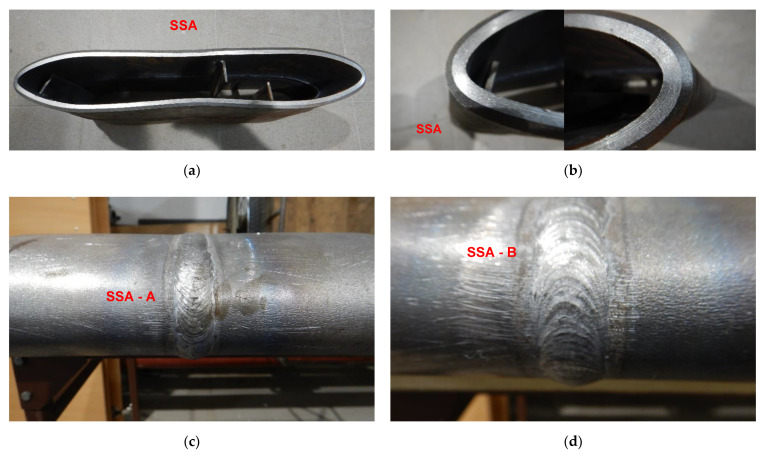
A view of SSA butt welded joint of API 5L X80 steel pipes and pipes edges after the 3000 kg impact loading test (the hammer height 1.0 m). No cracks of both extreme stressed/strained areas of the butt welded joint (**c**,**d**), and no cracks and tears of the extreme stressed/strained areas of the edges of the pipes (**a**,**b**).

**Figure 22:**
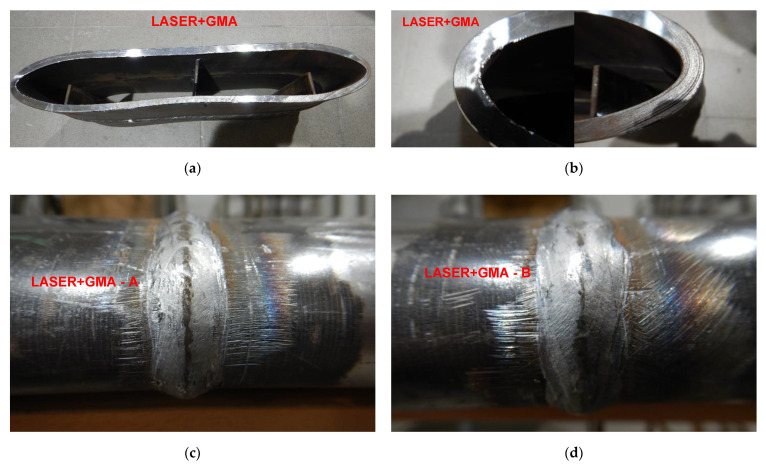
A view of LASER+GMA butt welded joint of API 5L X80 steel pipes and pipes edges after the 3000 kg impact loading test (the hammer height 1.0 m). No cracks of both extreme stressed/strained areas of the butt welded joint (**c**,**d**), and no cracks and tears of the extreme stressed/strained areas of the edges of the pipes (**a**,**b**).

To force failure (cracks) in extreme stressed/strained areas of the MMA butt welded joint, impact loading energy was increased by 25%, and the 3000 kg hammer height was increased to 1.5 m. After this second impact load test, the specimen was flattened to the plate shape, and both extremes stressed/strained areas of the MMA butt welded joint specimen cracked, but surprisingly no cracks appeared on the MMA welded pipes edges (Figure 17, Figure 18 and Figure 19). Results of impact loading test at 3000 kg hammer mass and the 1.0 m hammer height of GMA, SSA, and LASER+GMA butt welded joints of API 5L X80 pipes are shown in Figure 19, Figure 20, Figure 21 and Figure 22 and Table 7.

## 5. Conclusions

The FEM nonlinear analysis of the MMA, GMA, SSA, and LASER+GMA butt welded joints of API 5L X80 steel pipes, 323.9 mm dia. and wall thickness 10.0 mm of the resilience to catastrophic impact loading of welded joints of pipes by the hammer of the die forging hammer apparatus, Figure 14, indicates as follows:

1. The numerical simulations of the catastrophic impact loading of butt welded joints of pipes’ specimen have been performed by using the FEM modeling and the computer system LS-DYNA. The explicit analysis has been carried out, considering the pipes’ material properties and geometrical nonlinearities. Results of numerical simulations indicate that the weld metal + HAZ does not crack and also parent material or it is not submitted to any failure even though the Huber–Mises stresses and effective plastic strains are beyond the yield strength (R_e_ = 0.618 GPa) or even tensile strength (R_m_ = 0.700 GPa) of the weld metal and API 5L X80 steel parent material, Table 3, Table 5 and Table 6, Figure 6, Figure 7, Figure 8, Figure 9, Figure 10, Figure 11 and Figure 12. The dynamic fast-changing numerical simulation shows that the Huber–Mises stresses reach such values as 0.8 GPa for the impact loading hammer height H = 1.0 m and 0.95 GPa for the hammer height H = 1.5 m. The energy balance has been conducted to confirm the correctness of the obtained numerical results. The potential energy (Ep = m·g·h) corresponds approximately to the total energy for the final position of the butt welded joints of API 5L X80 steel pipes (after deformation) obtained from the presented graphs (Figure 8 and Figure 9) for both analyzed variants of the impact loading hammer height (H = 1.0 m and 1.5 m). From the data obtained using numerical calculations concerning the selected physical quantities, the following conclusions can be drawn:
  - Along with the increase of the height of the released hammer:
    ✓ the equivalent Huber–Mises stresses grow;
    ✓ the equivalent effective plastic strains grow;
    ✓ the resultant displacements grow;
  - Along with the increase of the height of the impact loading hammer, the thickness of the arc butt welded joints and wall thickness of pipes changes insignificantly which could be neglected.
  Finally, the height and width of the welded joints of pipes’ specimens after deformation obtained from the numerical calculations were compared with data received from experiments of hammer impact loading. The comparison of numerical and experimental results demonstrates a good agreement, proving that FEM simulation of technological processes is a useful tool to support experimental study.
2. The developed impact loading technique to evaluate the resilience to catastrophic failure of the MMA, GMA, SSA, and LASER+GMA butt welded joints of API 5L X80 steel pipes under simulated catastrophic impact loading events in refineries piping systems and proved to be very efficient and accurate. All impact loaded specimens of the MMA, GMA, SSA, and LASER+GMA butt welded joints of API 5L X80 steel pipes at impact energy forced by the hammer mass 3000 kg and at the hammer height H = 1.0 m, forcing at the extreme areas of butt welded joints the Huber–Mises stresses and effective plastic strains beyond the yield strength (R_e_ = 0.618 GPa) or even tensile strength (R_m_ = 0.700 GPa) of the weld metal and API 5L X80 steel pipes, proved high resilience to catastrophic impact loads, as no cracks or tears were detected, Table 1, Table 5 and Table 7, Figure 16 and Figure 19, Figure 20, Figure 21 and Figure 22.
3. Impact loaded specimens of MMA butt welded joints of API 5L X80 steel pipes at impact energy forced by the hammer mass 3000 kg and at the hammer height H = 1.5 m, forcing stresses and strains at the extreme areas of MMA butt welded joints and the parent material of pipes approximately 25% higher than the tensile strength of weld metals and parent material of pipes, Table 1 and Table 6, resulted in total flattening of the MMA butt welded joints of pipes specimens to H = 20 mm (double thickness of the pipes t = 10 mm). All welded joints strongly cracked at the extreme areas, but no cracks or tears appeared on the extreme edges of pipes, proving the very high quality of API 5L X80 steel pipes tested, Table 7, Figure 17, Figure 18 and Figure 22.

## Figures and Tables

**Table 1:** Mechanical properties and chemical composition %wt. of API 5L X80 high strength steel pipes 323.9 mm dia. and wall thickness 10.0 mm, produced by HUTA ŁABĘDY S.A. [12].

R_0.5_ MPa	R_m_ MPa	A5 %	KV at 0 °C J	C%	Si%	Mn%	Cr%	Ni%	Ti%	Al%	V%	Nb%
633	690	32	186–206	0.0766	0.232	1.33	0.2	0.019	0.0214	0.038	0.042	0.049

**Table 2:** The welding conditions used to produce specimens of butt welded joints of API 5LX80 steel pipes 323.9 diameter and wall thickness 10.0 mm.

Welding Process/Welding Position	Filler Metal/ Polarity	Joint Prep.*	Passes	Filler Metal dia. [mm]	Welding Current [A]	Welding Voltage [V]	Travel Speed [cm/min]	Heat Input [kJ/cm]
MMA/PC-2G	Conarc 85 DC+	α = 50−60° b = 2−4 mm, c = 2.0 mm	root	2.5	65−75	21.0−23.0	5.0−7.0	9.4−16.6
			filling	3.2	115−125	23.0−26.0	16.0−24.0	5.5−9.8
			cap	3.2	115−125	23.0−26.0	16.0−24.0	5.5−9.8
MMA/PH-3G			root	2.5	65−75	21.0−23.0	3.0−5.0	13.1−27.6
			filling	3.2	110−120	23.0−26.0	12.0−16.0	7.6−12.5
			cap	3.2	110−120	23.0−26.0	12.0−16.0	7.6−12.5
GMA/PC-2G	LNM MoNiVa Shielding gas M21-flow rate = 12−16 L/min DC+	α = 50−60° b = 2−4 mm, c = 2.0 mm	root	1.2	100−110	16.0−19.0	10.0−13.0	5.9−10.0
			filling		190−220	21.5−23.5	28.0−34.0	5.8−8.9
			cap		190−220	21.5−23.5	28.0−34.0	5.8−8.9
GMA/PH-3G			root	1.2	90−105	16.0−18.0	9.0−12.0	5.8−10.1
			filling		160−180	18.0−20.0	22.0−28.0	5.0−12.3
			cap		160−180	18.0−20.0	12.0−16.0	8.6−14.4
SSA/PC-2G	PIPELINER NR-208XP DC-	α = 50−60° b = 2−4 mm, c = 2.0 mm	root	2.0	110−120	16.0−19.0	8.0−12.0	7.0−13.7
			filling		180−200	21.0−24.0	25.0−35.0	5.2−9.2
			cap		180−200	21.0−24.0	25.0−35.0	5.2−9.2
SSA/PH-3G			root	2.0	110−120	16.0−19.0	7.0−9.0	9.4−15.6
			filling		150−170	20.0−23.0	15.0−20.0	7.2−12.5
			cap		150−170	20.0−23.0	15.0−20.0	7.2−12.5
Root pas welding	Filler metal		Joint prep.	Beam quality and beam focusing system		Beam power [kW]	Welding speed [m/min]	Heat input [kJ/cm]
Laser Yb:YAG TruDisk 12002 fiber = 300 µm	No filler metal, shielding gas–Ar 12.0 L/min gas nozzle dia.= 8.0 mm		α = 60o b = 6.0 mm, c = 0.0 mm	≤12.0 mm xmrad TRUMPF D70, fc = 200 mm, fcog = 400 mm, dcg = 0.8 mm		4.8	0.8	3.69

* Legend: *-α–bevel angle, b–root gap, c–root face. All joints preheat temperature–min 100 °C, interpass temperature-max. 250 °C.

**Table 3:** Material properties of the weld metals and API 5LX80 steel pipes being impact loaded in the die forging hammer apparatus.

Basic Material Properties	Symbol	Value
Young’s modulus	E	205 GPa
Poisson’s ratio	ν	0.28
Kirchhoff’s modulus	G	80 GPa
Tangent modulus	E_T_	0.558 GPa
Yield strength	R_0.2_	0.618 GPa
Ultimate tensile strength	R_m_	0.700 GPa

**Table 4:** The details concerning the individual parts of the physical model.

Parts	Type of Parts	Number of Elements	Number of Nodes
Pipe	Deformable	2000	2000
Hammer	Rigid	420	660
Base plate	Rigid	420	660
Weld metal	Deformable	200	300
Total in the model	-	3040	3620

**Table 5:** The juxtaposition of Huber–Mises stresses and effective plastic strains during impact loading of the specimens of welded joints of pipes for height H = 1.0 m, Figure 6.

No	Time [ms]	Huber–Mises Stress [MPa]	Effective Plastic Strain [mm/mm]
1.	373	403	0.000
2.	374	618	0.0019
3.	384	651	0.0587
4.	394	655	0.0668
5.	404	680	0.1110
6.	414	703	0.1519
7.	424	724	0.1908
8.	434	742	0.2225
9.	444	763	0.2597
10.	454	779	0.2873
11.	464	794	0.3155
12.	474	800	0.3256
13.	480	782	0.3260
14.	481	762	0.3260

**Table 6:** The juxtaposition of Huber–Mises stresses and effective plastic strains during impact loading of the specimens of welded joints of pipes for height H = 1.5 m.

No	Time [ms]	Huber–Mises Stress [MPa]	Effective Plastic Strain [mm/mm]
1.	491	426	0.0000
2.	492	619	0.0029
3.	502	647	0.0604
4.	512	676	0.1031
5.	522	708	0.1616
6.	532	743	0.2236
7.	542	785	0.2989
8.	552	835	0.3888
9.	562	894	0.4937
10.	572	940	0.5754
11.	578	760	0.5951
12.	579	623	0.5951

**Table 7:** Results of impact loading tests of MMA, GMA, SSA, and LASER+GMA butt welded joints of API 5L X80 pipes specimens.

Type of Joint	H [mm]	B [mm]	Quality of Welded Joint	Quality of Pipes Edges
MMA	74	456	no cracks	no cracks
MMA1	20	485	cracks	no cracks
MMA2	20	488	cracks	no cracks
GMA	75	466	no cracks	no cracks
SSA	78	463	no cracks	no cracks
LASER+GMA	55	474	no cracks	no cracks

## Data Availability

Not applicable.
